# Hair penalties: the negative influence of Afrocentric hair on ratings of Black women’s dominance and professionalism

**DOI:** 10.3389/fpsyg.2015.01311

**Published:** 2015-08-31

**Authors:** Tina R. Opie, Katherine W. Phillips

**Affiliations:** ^1^Management Division, Babson College, Babson ParkMA, USA; ^2^Management Division, Columbia Business School, New YorkNY, USA

**Keywords:** dominance, agency, women, professionalism, Black, hair, diversity, race

## Abstract

**Purpose:** Women are penalized if they do not behave in a stereotype-congruent manner (Heilman, 1983, 2001; Eagly and Carli, 2007). For example, because women are not expected to be agentic they incur an “agency penalty” for expressing anger, dominance or assertiveness (Rudman, 1998; Rudman and Glick, 1999, 2001; Eagly and Karau, 2002; Rudman and Fairchild, 2004; Brescoll and Uhlmann, 2008; Livingston et al., 2012). Yet, all women are not equally penalized (Livingston et al., 2012). We make a novel contribution by examining how both White and Black evaluators respond to displays of Black women’s dominance, in this case, whether Black women choose to wear Afrocentric or Eurocentric hairstyles.

**Design/methodology/approach**: We conducted three experimental studies to examine the influence of target hairstyle and participant race on ratings of the target’s professionalism (Studies 1, 2, and 3) and dominance (Study 2). Study 1 was an online experimental study with 200 participants (112 females, 87 males, 1 missing gender; 160 Whites, 19 Blacks, 11 Latinos, 7 Asian Americans and 3 who identify as “other”; *M*_age_ = 35.5, *SD* = 11.4). Study 2 was an online experimental study with 510 participants (276 women, 234 males; 256 Blacks, 254 Whites; *M*_age_ = 41.25 years, *SD* = 12.21). Study 3 was an online experimental study with 291 participants (141 Blacks, 150 Whites, *M*_age_ = 47.5 years, *SD* = 11.66).

**Findings**: Black, as compared to White, evaluators gave higher agency penalties to Black employment candidates when they donned Afrocentric versus Eurocentric hair, rating them as more dominant and less professional.

**Implications:** The present research illustrates the significance of considering both target and evaluator race when examining the influence of agency, and specifically dominance, on ratings of professionalism.

## Introduction

Women face biases that hinder their workplace advancement (Eagly and Carli, 2007). These biases may result from a perceived lack of fit between women’s stereotypical attributes and the attributes necessary to perform effectively in the workplace (Heilman, 1983, 2001). Underlying these biases are expectations that women should behave in a way that is congruent with these prescriptive stereotypes (Heilman, 2001; Eagly and Karau, 2002). When women do not behave in a stereotype-congruent manner, they are penalized and workplace advancement is inhibited (Heilman, 2001, 2012). For example, one challenge confronting women is that they incur an “agency penalty” for expressing anger, dominance, or assertiveness (Rudman, 1998; Rudman and Glick, 1999, 2001; Eagly and Karau, 2002; Rudman and Fairchild, 2004; Brescoll and Uhlmann, 2008; Livingston et al., 2012). Regardless of position in the company (e.g., entry-level position or a CEO role), when women convey anger they are conferred lower status and salary than men who express anger, and lower status and salary than women who do not express anger (Brescoll and Uhlmann, 2008). Yet, all women are not equally penalized. Livingston et al. (2012) illustrated that target race moderated the relationship between dominance and backlash effects (i.e., less social and economic consequence for counterstereotypical behavior, Rudman, 1998; Rudman and Glick, 2001; Rudman and Phelan, 2008). Specifically, because Whites perceive Black women as more dominant than White women there is less backlash against Black women when they display dominant behavior than against White women who display the same dominant behavior (Livingston et al., 2012).

Dominance refers to agentic behavior where an individual uses assertive, controlling, threatening, or forceful forms of influence (Ridgeway, 1987; Carli, 2001). One limitation of the research on agency penalties is that it has largely focused on White evaluators. To our knowledge, no previous research has investigated how Black evaluators respond to Black women’s dominance. This oversight is increasingly glaring due to the “browning of America” (Taylor and Pew Research Center, 2014), the increasing numbers of women in the workplace (United States Census Bureau, 2012), and the fact that Black women comprise one of the largest groups of working minority women (Department of Labor, 2013). To explore this neglected area with a growing and important population, we investigate how both White and Black evaluators respond to Black women’s expression of dominance. The consideration of both Black and White evaluators helps to address the critique that psychological research often lacks diversity in research samples (Sugden and Moulson, 2015). Further, we introduce a novel approach to examining dominance in the workplace.

It might be assumed that Black women would evaluate each other more positively than Whites evaluate them (Brewer, 1979, 1999; Tajfel and Turner, 1986; Ashforth and Mael, 1989; Hewstone et al., 2002) because of the classic finding that people appraise their in-group members more positively than out-group members (e.g., see Brewer, 1979; Hewstone et al., 2002; Balliet et al., 2014). Yet, there is a competing prediction that dominant Black women will receive a *higher* agency penalty from Black evaluators than from White evaluators.

Individuals are more attuned to differences amongst in-group members than they are amongst out-group members (Park and Rothbart, 1982; Messick and Mackie, 1989; Boldry et al., 2007), especially if the basis of the difference is negatively valenced (Rubin and Badea, 2007). This suggests that Black individuals may be particularly attuned to and judgmental of negative traits exhibited by other Black people. Dominance is a negatively valenced trait stereotypical of Blacks (Devine, 1989; Wittenbrink et al., 1997), thus, we posit that Black evaluators, more so than White evaluators, will be prone to penalize other Black individuals when it comes to dominance. Additionally, the concept of meta-stereotypes suggests that Black individuals’ beliefs about how they are perceived by White people may influence how Blacks respond to dominance (Vorauer et al., 1998). Blacks believe that Whites stereotype the Black race as dominant and aggressive (Sigelman and Tuch, 1997). Because Whites are a higher status group, they are considered to be better attuned to the competencies necessary for societal success (Vorauer and Sakamoto, 2008). If Blacks believe that dominance is a negative trait and that Whites consider Blacks to be dominant, Blacks may have heightened negativity toward Black women’s dominance because they think White people negatively stereotype such behavior. Blacks may have concerns that Black women’s dominance displays will reinforce negative stereotypes about Blacks (Duguid et al., 2012). As a result of these heightened concerns, Blacks may be unwilling to support (Duguid, 2011; Duguid et al., 2012), and perhaps they will even denigrate, Black women who display dominance.

Overall, because of in-group sensitivity, the fact that dominance is a negatively valenced trait stereotypical of Blacks (Devine, 1989; Wittenbrink et al., 1997) and that Blacks may have concerns about the reinforcement of negative stereotypes about Blacks, we posit that Black evaluators, more so than White evaluators, will be attuned to and more likely to penalize Black women’s dominance.

### Black Women’s Dominance

Prior research has examined reactions to women’s dominance primarily by exploring how participants respond to women’s behavior. For example, studies have examined the attributions participants make about women when they verbally convey anger versus sadness (Brescoll and Uhlmann, 2008); or, how participants respond to leaders when they have a dominant versus communal response to a subordinate employee’s failure to meet expectations (Livingston et al., 2012). But, dominance can be displayed in a number of ways, such as eye gaze (Mazur, 1985; Tang and Schmeichel, 2015), body posture (Carney et al., 2010; Rule et al., 2012), vocal pitch (Puts et al., 2007; Wolff and Puts, 2010), and facial expression (Knutson, 1996; Rule et al., 2012; Robinson et al., 2014). In our research we introduce another way to investigate dominance by exploring Black women’s choices about how they display their hair; that is, whether these women choose to wear their hair in a Eurocentric (i.e., straight) or Afrocentric (i.e., curly, kinky) manner^1^.

We use Optimal Distinctiveness Theory (ODT, Brewer, 1991) to explain why hair style choice can be a type of dominance display. ODT asserts that people actively manage the balance between the need for belongingness and the need for uniqueness. To accomplish this balance, people selectively choose from and activate social identities they perceive fit in the professional context (Pickett et al., 2002). Concerns about constructing a professional image and communicating belongingness in the professional context may cause those with marginalized identities to be particularly sensitive about how much, if any, of their marginal identity traits they reveal at work (Roberts, 2005). Indeed, professional image may be damaged if one reveals marginalized identity traits connected to race, ethnicity, sexual orientation, religion, gender or other marginalized categories (Thomas, 1993; Roberts, 2005). In an effort to protect their professional image, some individuals opt to downplay or conceal marginal identity traits such as an Afrocentric hairstyle (Ely, 1995; Roberts, 2005; Phillips et al., 2009; Shore et al., 2011; Shih et al., 2013) and instead wear the “mask of the professional” (McBroom, 1986) only allowing others to see their chosen professional identity (Roberts, 2005).

In the United States, this mask is likely to be constructed based on Eurocentric norms (Bell and Nkomo, 2003); hence, Black women who choose to wear Eurocentric hair styles may be conforming to this standard of professionalism in an effort to be accepted in the workplace, thereby fulfilling the need to belong. In contrast, when a Black woman chooses to wear an Afrocentric hairstyle she may be going against the norm revealing a marginal identity trait. This choice to go against the standard Eurocentric values may be thought of as an act of agency or dominance. The woman is not conforming to majority norms instead she is displaying a part of herself that is unique, fulfilling her need to feel distinct.

We also chose to focus on hair as a signal of dominance for three additional reasons. First, hair is a primary means to create, maintain, and transform identity (Weitz, 2001; Sheane, 2012) and is one of a host of traits used to determine one’s racial identity (Clair et al., 2005; Maddox, 2014; Pisares, unpublished dissertation thesis) being second only to skin in determining “Blackness” (Mercer, 1994). Given that dominance is a stereotype associated with Black people (Devine, 1989; Wittenbrink et al., 1997), the presence of Afrocentric hair may trigger the dominance stereotype. Second, unlike relatively immutable (racial) phenotypic traits such as skin color, eye color, nose width and lip size (Hill, 2002), hair texture is readily mutable. This makes hair an important identity trait to consider because Black women can choose whether to make their hair a visible or invisible racial characteristic (Clair et al., 2005). Third, Afrocentric hair has been associated with militancy and resistance (Davis, 1994; White, 2005; Bellinger, 2007) and militancy is a type of dominance. Thus, when Black women don Afrocentric hair, it may cause the stereotypes associated with Black people to become particularly salient.

Overall, because of concerns about optimal distinctiveness, the important role of Black women’s hair in their professional image construction, and the identity-relevance of hair, Black women’s choices about how they wear their hair may have important implications for perceived dominance.

Historically, Black women’s choices about how to wear their hair has been informed by societal pressures to adopt Eurocentric standards of straight hair (Lester, 2000). However, in the United States during the 1960s, Afrocentric hair began to be positively associated with the quest for equal rights in the Civil Rights and Black Power movements (Kelley, 1997). During this time, the slogan “Black is beautiful” was created to offset negative stereotypes about Black beauty (Hooks, 1995). Yet, by the 1970s the Afro had become largely masculinized in part because of its close association with the largely male, militant leadership of the Black Panthers (Kelley, 1997). Subsequently, there was a negative reaction to Black women wearing Afros. In particular, when worn by Black women, the Afro connoted a dominant woman who would engage in racial rebellion with employers and man-bashing to men. This created a dynamic where Black women began to experience pressure to straighten their hair (Kelley, 1997). Thus, since the 1970s, Black women donning Afros and other Afrocentric hairstyles in general, have been viewed as more dominant (and less feminine) than their Eurocentrically coifed counterparts. Further, Maddox’s (2014) work on racial phenotypicality indicates that individuals bearing Afrocentric traits were routinely denigrated and excluded from prestigious organizations and networks as a way to maintain social, economic, and educational structures that privileged White people created (Maddox, 2014). In summary, Black women who don Afrocentric hairstyles today may be viewed as more dominant, and therefore, less professional, than their counterparts who wear Eurocentric hairstyles.

In three studies, we explored whether Blacks, as compared to Whites, would more negatively rate Black women with Afrocentric hairstyles. Given the connection between Afrocentric hair and dominance, as well as, historical and contemporary stereotypes about Black women and Afrocentric hair, we predicted that all participants, regardless of race, would rate Eurocentric hairstyles more positively than Afrocentric hairstyles. We further predicted that Blacks, versus Whites, viewing Black women with Afrocentric hairstyles would rate the women as more dominant and therefore less professional. We also predicted that when qualitatively evaluating Black women with Afrocentric hairstyles, Black participants, as compared to White participants, would make more frequent mention of hair in response to a general, qualitative request for feedback on the job candidates.

### Overview and Hypotheses

In Study 1, we pursued initial evidence that employment candidates with Afrocentric hairstyles versus Eurocentric hairstyles would receive higher agency penalties and be rated as less professional. In Study 2, we examined how Black, as compared to White, individuals would rate employment candidates with Afrocentric versus Eurocentric hairstyles. Additionally, we investigated dominance as a potential mechanism underlying Black individuals’ reactions to Afrocentric hairstyles. We tested whether Black, as compared to White, individuals would rate employment candidates with Afrocentric hairstyles as more dominant and hence less professional than employment candidates with Eurocentric hairstyles. In Study 3, we tested the robustness of the race moderation effect. Further, we collected qualitative feedback from participants to assess whether participant race impacted the salience/importance of hairstyle when evaluating hypothetical job candidates. We wanted to ensure that attractiveness was not driving these results so we ran all of the below analyses with attractiveness as a covariate^2^ and the results were not affected. Throughout the paper, we report the analyses without attractiveness as a covariate. Additionally, outlier’s greater than three standard deviations were trimmed. Results with outliers trimmed are reported^3^. Finally, all research was carried out in accordance with the recommendations of Babson College’s Institutional Review Board. The protocol was approved by Babson College’s Institutional Review Board.

## Study 1

### Stimuli

**FIGURE 1:**
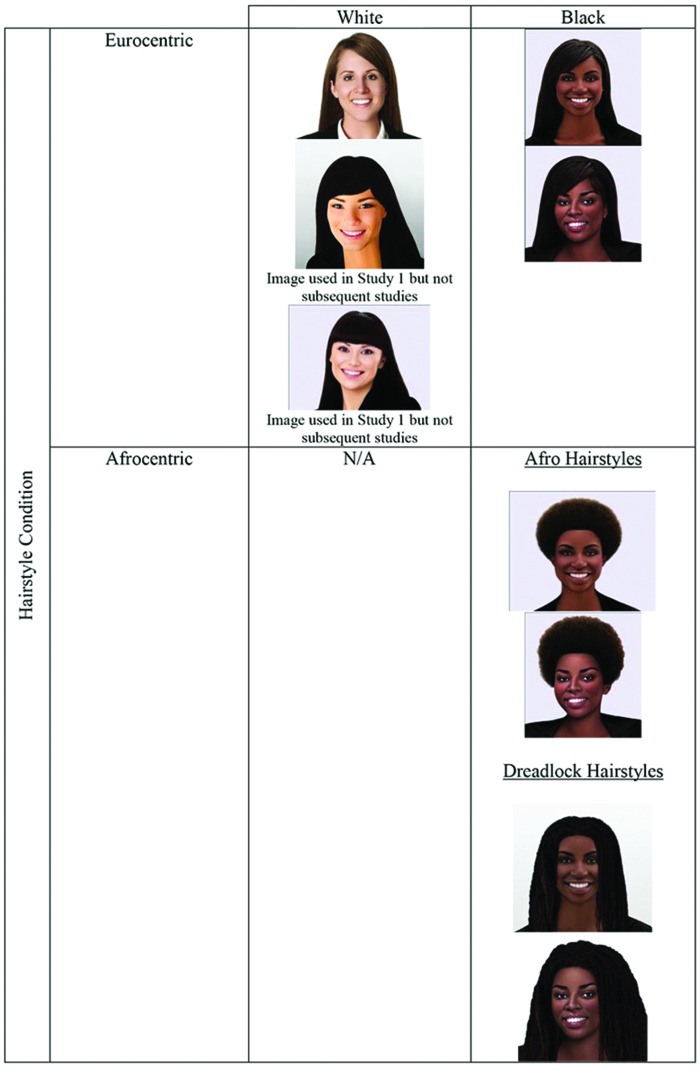
**Images used in studies**.

The first author and a professional Photoshop expert selected three images of White women and two images of Black women from iStockPhoto, an online photography provider, to enable subsequent testing of actor effects. All of the images were selected based on the actor having a facial position that was square with the camera and having a hairstyle that did not obstruct the shoulders so that the professional suit could be clearly viewed. The original hair was retained for two of the White female actors. We Photoshopped the hairstyle of the remaining White female actor so that all White female actors would have a similar straight hairstyle. Images of the Black females were Photoshopped so that there was a version of the two Black models with each of the following hairstyles: relaxed chemically processed (i.e., straight, Eurocentric hair), Afro hair, and Dreadlock hair (a hairstyle where hair grows into coiled ropes of hair). We chose two different Afrocentric hairstyles as this enabled us to investigate whether professionalism ratings were due to the overall Afrocentric category or a specific Afrocentric hairstyle. This resulted in nine versions of the stimuli (see **Figure 1**). Independent samples *t*-tests revealed no significant within-race actor or within hairstyle condition effects for the dependent variables of interest so we collapsed across hairstyle conditions resulting in Afrocentric and Eurocentric conditions. All of the *p*-values were greater than 0.1.

### Participants

The 200 participants (112 females, 87 males, 1 missing gender; 160 Whites, 19 Blacks, 11 Latinos, 7 Asian Americans and 3 who identify as “other”; *M*_age_ = 35.5, *SD* = 11.4, age range: 19–71) were recruited through Amazon M-Turk, an online survey participant platform. The survey was approximately 5 min long and participants were paid $1 for their participation.

### Procedure

After giving informed consent, each participant randomly viewed one of the stimuli and read:

*People regularly read personalities just from people’s faces. In this survey, you will see a head shot and answer various questions about the person’s personality based on their face*.

### Measures

#### Manipulation Check

Participants were asked, “What is the race/ethnicity of the person in this image? Please check the ONE that you think best matches.” Participants could choose from the following options: African–American/Black, Asian American, Latino(a), Caucasian/European–American, Native American, Middle Eastern, and Other.

#### Professionalism

We asked participants, “How professional is this person?” rated 1 (Very Unprofessional), 2 (Unprofessional), 3 (Somewhat Unprofessional), 4 (Average), 5 (Somewhat Professional), 6 (Professional) and 7 (Very Professional).

#### Corporate Success

We asked participants, “How likely is this person to succeed in Corporate America?”, rated 1 (Very Unlikely), 2 (Unlikely), 3 (Somewhat Unlikely), 4 (Average), 5 (Somewhat Likely), 6 (Likely) and 7 (Very Likely).

### Results

Thirteen of the participants failed to properly categorize the targets as White and Black. This resulted in 187 participants^4^. Prior research indicates that gender may play a role when evaluating targets of the opposite sex (e.g., see Eagly and Karau, 2002); therefore, we ran the analyses with and without gender as a covariate. The results remained the same and we report the analyses with gender as a covariate.

A Hairstyle^5^ (Afrocentric versus Eurocentric) ANCOVA with participant sex as a covariate (male, female) on evaluation of the employment candidate’s professional appearance yielded a significant main effect, *F*(1,183) = 36.23, *p* < 0.01, $\eta_{p}^{2}$ = 0.17. Pairwise comparisons using Bonferroni-adjusted alpha levels revealed the predicted patterns. Specifically, participants perceived the employment candidates with Afrocentric hairstyles as less professional than employment candidates with Eurocentric hairstyles, *t*(172) = 5.96, *p* < 0.001. Importantly, the mean professionalism ratings did not differ between the Afrocentric hairstyle conditions (i.e., Afro and Dreadlocks), *t*(93) = 0.004, *ns*. Nor did the mean professional rating differ between the Eurocentric hairstyle conditions (i.e., straight hair on the Black women and straight hair on the White women), *t*(89) = 1.46, *ns.* (see **Table 1**).

**Table 1:** Ratings of professional appearance and likelihood of success (Study 1).

	Hairstyle condition					

	Afrocentric			Eurocentric		
	Afro	Dreadlock	Afrocentric mean	Straight-Black	Straight-White	Eurocentric mean
Professional appearance	4.93^a^ (1.03)	4.93^a^ (1.10)	4.93^a^ (1.06)	5.87^b^ (0.70)	5.63^b^ (0.82)	5.74^b^ (0.77)
Likelihood of success	4.60^a^ (1.25)	4.78^a^ (1.51)	4.70^a^ (1.4)	5.87^b^ (0.98)	5.60^b^ (0.83)	5.71^b^ (0.90)

*Means with different letters differ significantly at *p* < 0.05.*

A Hairstyle (Afrocentric, Eurocentric) ANCOVA with participant sex as a covariate (male, female) on evaluation of employment candidate’s likelihood of success in Corporate America yielded a significant main effect, *F*(1,184) = 36.19, *p* < 0.01, $\eta_{p}^{2}$ = 0.16. Again, pairwise comparisons using Bonferroni-adjusted alpha levels revealed the predicted patterns. Specifically, participants perceived the employment candidates with Afrocentric hairstyles as less likely to succeed in Corporate America than employment candidates with Eurocentric hairstyles, *t*(163) = 5.87, *p* < 0.001. Importantly, the mean ratings of the likelihood of succeeding in Corporate America did not differ between the Afrocentric hairstyle conditions, *t*(94) = -0.65, *ns*. Nor did the mean ratings of the likelihood of succeeding in Corporate America differ between the Eurocentric hairstyle conditions, *t*(89) = 1.46, *ns*. (see **Table 1**).

### Discussion

The Study 1 results indicate that employment candidates with Afrocentric hairstyles were rated as less professional and less likely to succeed in Corporate America than employment candidates with Eurocentric hairstyles. Further, the only statistically significant differences were between those with Afrocentric or Eurocentric hairstyles as no within-condition differences were found. That is, the race of the candidate was less important than the Afrocentric or Eurocentric nature of the hairstyle as there was no difference in judgments of White candidates with straight hair and Black candidates with straight hair. Furthermore, both of the Eurocentric hairstyle images of the Black women were Photoshopped while only one of White images was manipulated. This did not appear to cause any systematic differences as there were no differences in ratings between the Black and White Eurocentric styles. Also, while the images were largely effective, some of the White female images had higher manipulation check failures. As a result, these images were not used in subsequent studies. Overall, the results provide preliminary support those employment candidates with Afrocentric hairstyles are indeed rated as less professional and less likely to succeed than employment candidates with Eurocentric hairstyles.

## Study 2

In Study 1, due to a largely homogenous sample, we were unable to compare Black and White participant responses to Black women’s dominance display. Thus, Study 2 was designed to test the relationship between hairstyle and ratings of professionalism, as well as, the participant race moderation effect amongst Black and White women and men. Additionally, given the historical meaning affixed to Afrocentric hair for Blacks, we examined a potential mediating mechanism, dominance, to explain why Black participants might react to Afrocentric hairstyles more negatively than Eurocentric hairstyles.

### Participants

We recruited 510 Black and White, male and female participants from CINT (an online panel provider) for a 15–20 minute study on “Reading Personalities from People’s Faces” in exchange for $5. Participants were comprised of 276 women, 234 males; 256 Blacks, 254 Whites; *M*_age_ = 41.25 years, *SD* = 12.21, age range: 18–64). In terms of employment status, 85% of participants were employed and the remaining 15% of the participants had been employed in the last 5 years.

### Materials and Procedure

The same stimuli used in Study 1 were used in Study 2, except failures (see **Figure 1**). After giving informed consent, each participant randomly viewed one of the stimuli and read the same instructions as in Study 1.

### Professionalism: Negativity about Candidate’s Appearance

To assess professionalism, we measured participants’ ratings of the candidate’s appearance using three items developed to reflect the lack-of-fit model (Heilman, 1983, 2001). Participants read, “Please indicate how much you agree or disagree with the following statements”: (a) “This employment candidate’s appearance is inappropriate for corporate America”; (b) “This employment candidate’s appearance does not fit corporate America”; (c) This candidate’s appearance will be problematic in corporate America. Response options were on a seven-point scale from 1 (Strongly Disagree) to 7 (Strongly Agree). Higher scores indicated more negative perceptions of the employment candidate, Cronbach’s α = 0.79.

### Dominance

Based on prior race and gender stereotype research (Wittenbrink et al., 1997; Fiske et al., 2002; Prentice and Carranza, 2002; Rudman et al., 2012), we asked participants to evaluate the employment candidate’s dominance based on how much they saw her to be “controlling,” “forceful,” “aggressive,” “dominating,” “threatening,” “ruthless,” and “intimidating.” Participants read, “Please rate the employment candidate on the following traits. We are not interested in your personal beliefs, but in how you think the individual is viewed by others.” We asked participants to indicate how they think the employment candidate is viewed by others to minimize the influence of social desirability bias (Fisher, 1993). Response options were on a seven-point scale from 1 (Not at all) to 7 (Extremely). Higher scores indicated higher dominance perceptions, Cronbach’s α = 0.90.

### Results

Independent samples *t*-tests on professionalism and dominance revealed no significant differences between the candidates with Eurocentric hairstyles (i.e., Black candidates with straight hair and White candidates with straight hair) as in Study 1; therefore, we collapsed across conditions resulting in one Eurocentric hairstyle condition (all *p*-values > 0.2). Independent sample *t*-tests revealed that there was a significant difference between the Afro (*M* = 2.60, *SD* = 1.55) and Dreadlock conditions (*M* = 3.14, *SD* = 1.70), *t*(272) = -2.76, *p* < 0.05 for the professionalism dependent variable. For dominance, the Afro condition (*M* = 2.69, *SD* = 1.33) was marginally significantly different from the Dreadlock hairstyles (*M* = 2.97, *SD* = 1.34), *t*(271) = -1.75, *p* = 0.08. Despite these differences among Afrocentric hairstyles, we collapsed across the Afro and Dreadlock hairstyles resulting in one Afrocentric condition. Importantly analyses not presented below indicate that the two Afrocentric hairstyles independently differ from the Eurocentric hairstyles with all *p*-values less than 0.001.

#### Manipulation Check

Participants were again asked to identify the race of the target. All participants answered correctly.

#### Professionalism: Negativity about Candidate’s Appearance

**FIGURE 2:**
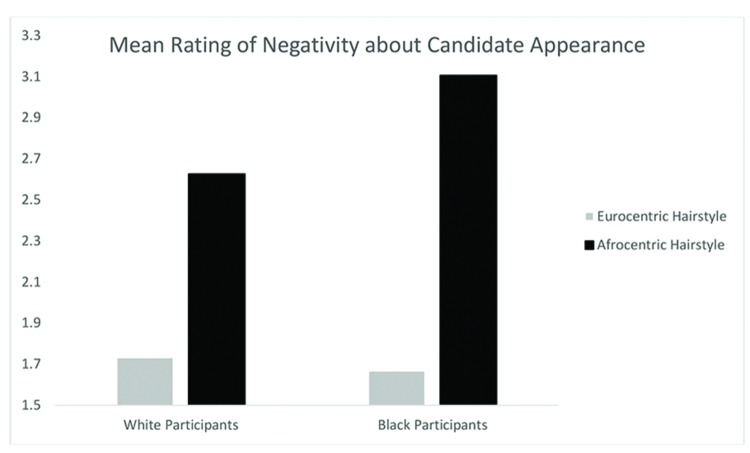
**Mean ratings of negativity about candidate appearance by participant race and hairstyle condition in Study 2**.

A Participant Race (Black, White) by Hairstyle (Eurocentric, Afrocentric) ANCOVA with participant sex as a covariate (male, female) on evaluation of employment candidates yielded a significant main effect for hairstyle, *F*(1,498) = 90.37, *p* < 0.01, $\eta_{p}^{2}$ = 0.15 such that the employment candidates with Afrocentric hair were rated significantly more negatively than those with Eurocentric hairstyles, as in Study 1. The main effect of participant race was significant, *F*(1,498) = 4.07, *p* < 0.05, with Black participants rating employment candidates more negatively than White participants, *t*(501) = -2.05, *p* < 0.05. Most importantly, the interaction effect was significant, *F*(1,498) = 5.05, *p* < 0.05, $\eta_{p}^{2}$ = 0.01 indicating that the effect of Afrocentric versus Eurocentric hairstyle was stronger amongst Black rather than White participants (see **Figure 2**). Black participants rated employment candidates with Afrocentric hair more negatively than employment candidates with Eurocentric hair, *t*(250) = -7.71, *p* < 0.001. For White participants there was still a difference, with Afrocentric hair being rated more negative than Eurocentric hair, *t*(249) = -5.78, *p* < 0.001, but the effect was not as strong. Finally, Black participants rated Afrocentric hairstyles more negatively than Whites rated the Afrocentric conditions, *t*(271) = -2.62, *p* < 0.01. Importantly, these effects were significant when participant ratings of the employment candidates’ attractiveness was entered as a covariate. This suggests that the influence of hairstyle and participant race on professionalism happens above and beyond perceptions of attractiveness (see **Table 2**).

**Table 2:** Ratings of professionalism: negativity about candidate’s appearance (Study 2).

Hairstyle condition			
Evaluator race	Afrocentric mean	Eurocentric mean	Total
White	2.59^a^ (1.42)	1.71^c^ (0.89)	2.19 (1.28)
Black	3.10^b^ (1.78)	1.68^c^ (0.94)	2.46 (1.62)
Total	2.85 (1.63)	1.70 (0.91)	2.32 (1.47)

*Means with different letters differ significantly at *p* < 0.05.*

#### Dominance

A Participant Race (Black, White) by Hairstyle (Eurocentric, Afrocentric) ANCOVA with participant sex as a covariate (male, female) on evaluation of employment candidates yielded no main effect for hairstyle on dominance ratings, *F*(1,500) = 1.40, *p* = 0.24 or for participant race on dominance ratings, *F*(1,500) = 0.01, *p* = 0.93. However, there was a significant interaction effect for participant race and hairstyle, *F*(1,500) = 4.94, *p* < 0.05, $\eta_{p}^{2}$ = 0.01 that supported our hypothesis. Black participants rated employment candidates with Afrocentric hair as more dominant than employment candidates with Eurocentric hair, *t*(251) = -2.32, *p* < 0.05. In contrast, White participants did not differ in their dominance ratings of employment candidates with Afrocentric hair versus Eurocentric hair, *t*(250) = 0.72, *ns*. (see **Table 3**).

**Table 3:** Ratings of dominance (Study 2).

Hairstyle condition			
Evaluator Race	Afrocentric mean	Eurocentric mean	Total
White	2.67^c^ (1.10)^a^	2.78^c^ (1.26)^a^	2.72 (1.18)
Black	2.92^a^ (1.45)^b^	2.51^b^ (1.36)^c^	2.73 (1.42)
Total	2.80 (1.29)	2.64 (1.32)	2.75 (1.31)

*Means with different letters differ significantly at *p* < 0.05.*

**FIGURE 3:**
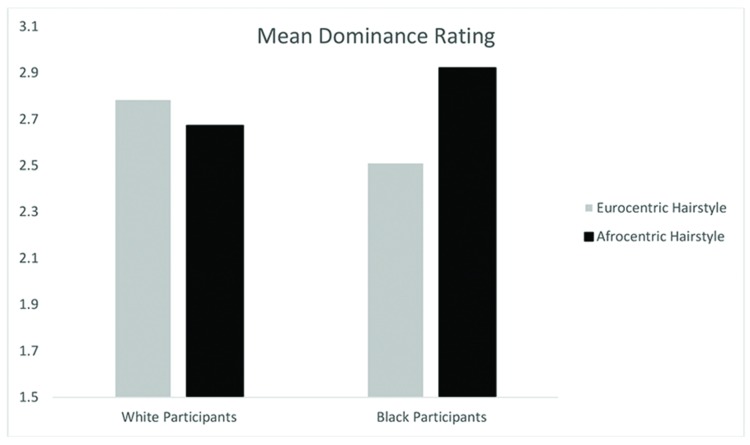
**Mean dominance rating by participant race and hairstyle condition in Study 2**.

Further, we examined how Black participants rated only Black candidates to isolate the contrast between Blacks with Afrocentric hair and Blacks with Eurocentric hair. Black participants marginally rated Black candidates with Afrocentric hair (*M* = 2.92, *SD* = 1.45) as more dominant than Black candidates with Eurocentric hair (*M* = 2.59, *SD* = 1.32), *t*(212) = -1.65, *p* = 0.1 (see **Figure 3**).

#### Mediation

To investigate whether dominance mediated the effect of hairstyle on professionalism ratings, we conducted mediation analyses using SPSS macro (PROCESS) for bootstrapping indirect effects (Preacher and Hayes, 2008). The macro estimates all paths using ordinary least squares regression. We used 10,000 samples to calculate the point estimates of both the indirect effects as well as the bias-corrected confidence intervals (CI; see Preacher and Hayes, 2004). Consistent with our predictions, for Black participants but not White participants, perceived dominance of the employment candidate mediated the effect of Afrocentric hairstyle on professionalism ratings of the employment candidates. Specifically, for White participants the point estimate was -0.03, SE of 0.04, and the 95% CI was [-0.11, 0.041]. For Black participants, the point estimate was 0.09, SE of 0.05 and the 95% CI was [0.02, 0.205]. While the 95% CI of the indirect effect of Afrocentric hairstyle on participants’ professionalism ratings of the target included zero for White participants, the CI did not include zero for Blacks participants. This indicates that perceived dominance was a significant mediator only for Black participants. Thus, our prediction was supported.

### Discussion

In Study 2, we observed that Afrocentric hair caused Black, more so than White, participants to rate the employment candidate as less professional. Further, for Black participants only, the relationship between Afrocentric hair and professional appearance was mediated by dominance perceptions. Overall, the results provide support for the argument that employment candidates with Afrocentric hairstyles are rated as less professional than employment candidates with Eurocentric hairstyles and evaluator race moderates this relationship.

## Study 3

Having indicated that Afrocentric hairstyles are rated as less professional than Eurocentric hairstyles (Studies 1 and 2); that participant race moderates this negative relationship; and that, for Blacks, dominance mediates this relationship (Study 2), Study 3 was designed to test the robustness of the participant race moderation effect. Further, we collected qualitative feedback from participants to assess whether participant race affected the salience of hairstyle when evaluating job candidates. Finally, given cross-gender rating implications (e.g., see Eagly and Karau, 2002) and theoretical interest in the unique in-group experiences of Black women, we only recruited Black and White women to participate in Study 3.

### Participants and Study Design

We recruited 291 Black and White women to participate in Study 3 (141 Blacks, 150 Whites, *M*_age_ = 47.5 years, *SD* = 11.66, age range: 18–64) from CINT (an online panel provider). Participants completed the 20-min study in exchange for a $5 payment. At the time of the study, 77% of the participants were employed, the rest had been employed in the past 5 years. Participants’ employment status did not affect the dependent measures in this study (all *p*-values are greater than.2), thus it will not be discussed further. The same stimuli and procedure used in Study 2 were used in this study. After giving informed consent, each participant randomly viewed one of the seven stimuli (see **Figure 1**) and read the same instructions as in Study 2.

### Measures

#### Professionalism: Negativity about Candidate’s Appearance

The same items used in Study 1 were used in this study; Cronbach’s α = 0.86.

#### Qualitative Assessments

After responding to scale items, participants were asked to answer three open-ended questions: (1) What advantages do you think the candidate has when seeking employment?; (2) What disadvantages do you think the candidate has when seeking employment?; and, (3) What advice would you give to the candidate to increase his/her chances of being hired? Please note that hair was not specifically mentioned in the questions because we wanted to ascertain how salient hair would be to the participants. We hypothesized that hair would be most salient to Black participants rating job candidates with Afrocentric hairstyles. Responses received a code of “1” if hair was mentioned in a positive way, “-1” if hair was mentioned in a negative way and “0” if there was no mention of hair.

### Results

#### Manipulation Check

All of the participants except thirteen individuals correctly reported the manipulation checks for target race (11 provided incorrect answers and two failed to answer the question; therefore, these 13 participants were eliminated from the analysis). Independent samples *t*-tests revealed no differences between the Black images; therefore, conditions were collapsed (all *p*-values are greater than 0.8). An independent samples *t*-test also revealed no differences between the Afrocentric hairstyle conditions and the Eurocentric straight hairstyle conditions; therefore, conditions were collapsed (all *p*-values are greater than 0.8).

### Quantitative Analysis

#### Professionalism: Negativity about Candidate’s Appearance

**FIGURE 4:**
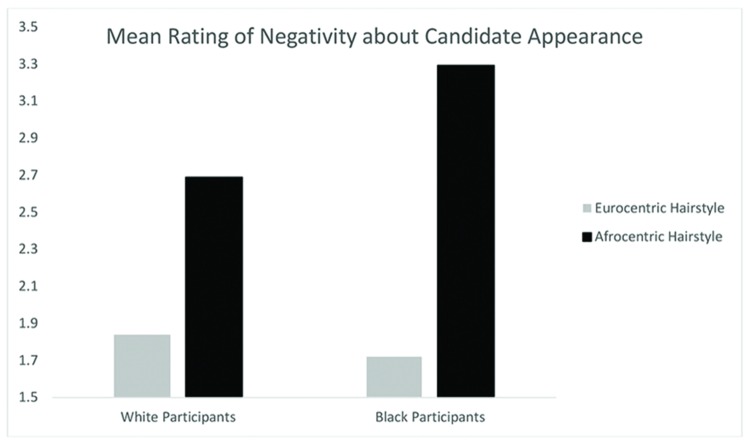
**Mean ratings of negativity about candidate appearance by participant race and hairstyle condition in Study 3**.

A Hairstyle (Afrocentric, Eurocentric) by Participant Race (Black, White) ANOVA yielded a main effect for Afrocentric hairstyle, *F*(1,271) = 60.22, *p* < 0.001, $\eta_{p}^{2}$ = 0.18, such that negative assessments of the employment candidate were significantly higher in the Afrocentric condition than in the Eurocentric condition, *t*(273) = -7.58, *p* < 0.001. The main effect of participant race was not significant, *F*(1,271) = 1.51, *p* = 0.22. However, the interaction between hairstyle and participant race was significant, *F*(1,271) = 6.28, *p* < 0.05, $\eta_{p}^{2}$ = 0.02 indicating that the Afrocentric effect was strongest amongst Blacks (see **Figure 4**). Black participants rated employment candidates with Afrocentric hair more negatively than they rated the employment candidates with Eurocentric hair, *t*(131) = -6.80, *p* < 0.001 and more negatively than White participants rated the Afrocentric conditions, *t*(155) = -2.55, *p* < 0.05. As in Study 2, for White participants there was still a difference, with Afrocentric hair being rated more negative than Eurocentric hair, *t*(140) = -3.97, *p* < 0.001, but the effect was not as strong as that found for Black participants. Black and White participants did not differ in negativity toward the employment candidates with Eurocentric hair *t*(116) = 1.04, *ns*. Thus, our predictions were supported (see **Table 4**).

**Table 4:** Ratings of professionalism: negativity about candidate’s appearance (Study 3).

Hairstyle condition			
Evaluator race	Afrocentric mean	Eurocentric mean	Total
White	2.69^a^ (1.38)	1.84^b^ (1.07)	2.33 (1.32)
Black	3.29^c^ (1.59)	1.63^b^ (1.10)	2.57 (1.62)
Total	2.98 (1.51)	1.74 (1.08)	2.45 (1.48)

*Means with different letters differ significantly at *p* < 0.05.*

### Qualitative Analysis

We next analyzed qualitative data collected for the same study to determine if participant feedback would also reflect Black participant’s heightened attention to and more negative evaluation of Afrocentric hairstyles, as compared to White participants. First, a chi-square test of independence was performed to examine the relation between hairstyle condition (i.e., Afrocentric and Eurocentric) and the frequency of hair being described as a disadvantage for the employment candidate. The relation between these variables was significant χ^2^(1, *N* = 284) = 33.45, *p* < 0.001. Participants were more likely to mention Afrocentric hair as a disadvantage as compared to Eurocentric hair. Interestingly, in the Eurocentric condition, hair was not mentioned as a disadvantage even one time (**Table 5**).

**Table 5:** Frequencies of hair mentioned as a disadvantage by hairstyle condition (Study 3).

Disadvantage for employment candidate	Hairstyle condition		
	Afrocentric hairstyle	Eurocentric hairstyle	χ^2^
Hair mentioned in a negative way	37	0	33.45
Hair not mentioned	122	125	

We also found a significant relationship between hairstyle condition and advice, χ^2^(1, *N* = 278) = 38.13, *p* < 0.001. Participants were more likely to provide advice that mentioned hair in a negative way in the Afrocentric condition than in the Eurocentric condition. Again, in the Eurocentric condition, hair was not mentioned in a negative way even one time (**Table 6**). There was no significant relationship between hairstyle condition and advantage, χ^2^(1, *N* = 283) = 1.30, *p* = 0.25.

**Table 6:** Frequencies of advice mentioning hair by hairstyle condition (Study 3).

Advice for employment candidate	Hairstyle condition		
	Afrocentric hairstyle	Eurocentric hairstyle	χ^2^
Hair mentioned in a negative way	42	0	38.13
Hair not mentioned	115	121	

We next performed a chi-square test of independence amongst the Afrocentric hairstyles to examine the relation between participant race and the frequency of hair being described as a disadvantage for the employment candidates with Afrocentric hairstyles. The relation between these variables was significant χ^2^(1, *N* = 159) = 8.05, *p* = 0.005 (**Table 7**). Black participants, as compared to White participants, were more likely to mention Afrocentric hair as a disadvantage. Here are a few direct quotations from the Afrocentric condition illustrating hair as a disadvantage (quotations from Black participants):

**Table 7:** Frequencies of hair as a disadvantage within Afrocentric condition by participant race (Study 3).

Disadvantage for employment candidate	Participant race		
	Black participants	White participants	χ^2^
Hair mentioned in a negative way	25	12	8.05
Hair not mentioned	50	72

(1) “People judging her based on her looks, particularly her hair.”
(2) “Her natural hair style would probably be intimidating to caucasion employers”
(3) “Some people may not like her hairstyle”
(4) “Her hair should change”
(5) “I think her all natural hair style will create a problem for some employers.”

Here are a few direct quotations from the Afrocentric condition where hair is not mentioned as a disadvantage (quotations from Black participants):

(1) “She is Black and she is a woman. Plus she appears rather young, so that might read as inexperienced.”
(2) “No experience, lacking of education background, not professional, and always coming to work late.”
(3) “She is african american and female”
(4) “Masculine attire, too much eye contact”
(5) “No disadvantages at all.”

Further, for Afrocentric hairstyles there was no significant relationship between participant race and advice χ^2^(1, *N* = 157) = 1.12, *p* = 0.29 or between participant race and advantage, χ^2^(1, *N* = 159) = 1.81, *p* = 0.18.

Finally, we performed a chi-square test of independence amongst the Eurocentric hairstyles to examine the relations between participant race and the frequency of hair being described as a disadvantage for the employment candidates with Eurocentric hairstyles. The relation between these variables was not significant, χ^2^(1, *N* = 124) = 1.03, *p* = 0.31. We were unable to calculate a chi-square for the relation between Eurocentric hair and advantage or Eurocentric hair and advice because in each instance, hair was never described in a negative way. Interestingly, hair was not mentioned even one time in the advice for the employment candidates with Eurocentric hairstyles.

### Discussion

Study 3 provides further evidence that employment candidates with Afrocentric hairstyles are rated as less professional than employment candidates with Eurocentric hairstyles regardless of evaluator race. Yet, this study provides additional evidence that evaluator race matters since Black women, as opposed to White women, have *more* negative perceptions of how Afrocentric hairstyles will be perceived in the workplace. In this study, we also used attractiveness as a covariate and the significant effects remained. This suggests those Black participants’ more negative ratings of employment candidates with Afrocentric hair happens above and beyond perceptions of attractiveness. Qualitative analysis supported our quantitative findings. Black individuals, as compared to White individuals, were more attuned to dominance displays of Black women making more frequent mention of Afrocentric hair as a disadvantage for the Black employment candidates. Thus, our predictions were supported.

## General Discussion

The present research illustrates the significance of considering both target and evaluator race when examining the influence of agency, and specifically dominance, on perceptions of professionalism. Our results are consonant with past research, as we found evidence of an agency penalty against targets who displayed dominance (Livingston et al., 2012). Yet, our novel contribution was the evidence that evaluator race influences dominance perceptions and subsequent ratings of professionalism. Further, we introduced a new way, hairstyles, to investigate dominance in the workplace. We used ODT to explain why hair is a signal of dominance. ODT asserts that people need to maintain balance between the need for belongingness and the need for uniqueness. In an effort to achieve this balance, people select from and activate various identities that they will reveal in the workplace (Pickett et al., 2002). Yet, because of concerns about maintaining a professional image, there is pressure to suppress marginalized identity traits (e.g., traits connected to race, gender, etc.; Thomas, 1993; Roberts, 2005). In other words, those with marginalized identity traits may suppress those traits (hence suppressing their uniqueness) in order to fulfill a need for belongingness. Hence, Black women’s decisions to reveal a marginal identity trait (i.e., Afrocentric hair) in the workplace can be considered an act of dominance because they are opting not to comply with majority norms but rather asserting their uniqueness.

In Studies 1, 2, and 3, participants randomly assigned to evaluate employment candidates rated the candidates with Afrocentric hair as less professional than candidates with Eurocentric hair, regardless of participant race. As predicted, for Black participants, dominance mediated the relationship between hairstyle and professionalism ratings, such that employment candidates with Afrocentric hairstyles were rated as more dominant and hence less professional. White participants did not demonstrate this mediated effect perhaps because as members of the out-group they are not as attuned to or sensitive to the perception that Black women’s hair can be seen as a dominance display. In line with this conclusion, the results of Study 3 showed that White participants, as compared to Black participants, less frequently mentioned hair as a disadvantage for the Afrocentric candidates.

### Theoretical Contributions

Our current findings suggest several theoretical implications for the literature on diversity, agency penalties, and dominance. First, in contrast to classic research which has found that people appraise their in-group members more positively than out-group members (e.g., see Brewer, 1979; Hewstone et al., 2002; Balliet et al., 2014), our findings indicate that Black evaluators, as compared to White evaluators, ascribe a *higher* agency penalty to Black women when they display dominance. This suggests that Blacks do not show in-group favoritism about an in-group identity trait (i.e., Afrocentric hair) that relates to dominance. In fact, we theorize that Black perceivers will not show in-group favoritism. Afrocentric hair directly links to negative stereotypes about Black people (Maddox, 2014). When Black women don Afrocentric hair, Black perceivers may have heightened concerns that the Black women’s dominance display will negatively reflect on all Blacks (Duguid et al., 2012). This helps to explain why Black evaluators, as compared to White evaluators, would assess a higher agency penalty for dominant Black women.

Second, the idea that in-group members of marginalized groups may more negatively evaluate in-group identity traits has important implications for diversity research. Much research on diversity and inclusion focuses on how powerful decision-makers can be encouraged to create workplaces that are more hospitable and accepting of marginalized group members. Yet, our research indicates that historical context and negative stereotypes associated with marginalized social identity traits may prompt in-group members to penalize each other for displaying these identity traits. In turn, such penalties may detract from marginalized group members’ willingness to express these traits in the workplace. Unfortunately, workplace diversity may be hindered if marginalized individuals are reluctant to express their identity traits in the workplace.

Our research contributes to a growing body of research concerned with diversity and identity management in organizations. While Afrocentric hairstyles may be devalued by both Whites and Blacks, we find evidence that Black people are the most negative in their evaluation of Afrocentric hairstyles. We suggest that in addition to acquiescing to external pressure from powerful decision-makers to adhere to Eurocentric professional standards, Black individuals may also be conforming to in-group standards about what is and is not professional. We believe that this in-group dynamic is not specific to Black women and Afrocentric hair and that other minority in-group members may also impose covering “demands” on their members: gay people are expected to signal that they are gay without appearing “too gay” and women must be both feminine and masculine depending upon the context (Robinson, 2007). Research on horizontal hostility also provides supporting evidence that in-group members indeed police each other about adherence to in-group notions of what is and is not acceptable in-group behavior (White and Langer, 1999; White et al., 2006). Our research indicates that the in-group can penalize other minority in-group members if they fail to adhere to such covering demands. Thus, we posit that our research may tap into a more general phenomenon that can inform further inquiry into the in-group dynamics of an array of demographic categories.

Below, we elaborate on why marginalized individuals may be disinclined to display identity traits that signal membership in marginalized demographic categories. This discussion yields several important practical implications for organizations.

### Practical Implications

Organizations strive to create diverse workplaces at least in part because of diversity’s positive relationship with performance (see Mannix and Neale, 2005 for a review). Our research suggests that as organizations endeavor to diversify their workforces, they may also need to consider the challenges confronting employees who opt to express marginalized identity traits. While it is understandable that people quickly form first impressions of others based on appearance (Willis and Todorov, 2006; Olivola and Todorov, 2010), the challenge here is that those first impressions influence ratings of perceived dominance and professionalism. Consequently, organizations may encourage diversity but be unaware that employees who display marginalized identity traits indicative of that diversity may find themselves at a disadvantage compared to employees who suppress these traits.

In the case of Black women and Afrocentric hair, Black people seem well aware of the disadvantages they may reap if they display their Afrocentric hair. Black women’s awareness of the disadvantages of Afrocentric hair was palpable in the Study 3 qualitative results where Black women repeatedly mentioned that Afrocentric hair was a disadvantage in the workplace. These disadvantages may arise because Afrocentric hair makes race salient (Maddox, 2014) which may trigger negative stereotypes of Blacks. In turn Blacks may engage in social identity-based impression management strategies (i.e., deemphasize social identity traits that signal group membership) to manage the impact of these negative stereotypes on others’ perceptions of their competence and character (Roberts, 2005). Concerns about professional image construction may lead Black women to conform to Eurocentric appearance standards and suppress identity traits.

Unfortunately, the suppression of identity traits may have harmful effects for both individuals and organizations. Individuals may experience lower commitment to their organization and feelings of alienation (Yoshino and Smith, 2013), as well as, heavier emotional and cognitive burdens as they ponder how best to manage how they are perceived by others (Rosette and Dumas, 2007; Opie, 2015). At the organizational level, individuals’ identity suppression may deprive organizations of the richness that diversity affords. Additionally, organizations may suffer from litigation if employees negatively react to perceived organizational requirements to suppress their identity traits.

While we have discussed the practical implications for organizations and individuals with marginalized identity traits, we now turn to a specific discussion about the unique implications for Black people and their display of marginalized identity traits, in this case, Black women’s Afrocentric hair. We have argued that Afrocentric hairstyles, as compared to Eurocentric hairstyles, will be rated as less professional regardless of evaluator race. We found support for this prediction. Additionally, we predicted and found evidence that evaluator race moderates the relationship between Afrocentric hairstyle and ratings of professionalism. We posit that this race moderation effect occurs because Black people are sensitized to displays of Afrocentric hair due to the stereotype-relevant nature of Afrocentric hair. That is, Afrocentric hair is directly linked to stereotypes about Black people (Maddox, 2014). Therefore, if Black women don Afrocentric hair other Black perceivers may believe that they will be negatively judged because of the display of Afrocentric hair (Duguid et al., 2012). This concern about negative judgment may lead Black people, as compared to White people, to be particularly harsh when judging Black women with Afrocentric hair as we found in our studies.

Our discussion about why marginalized individuals may suppress social identity traits may create the impression that organizations have less than desirable levels of diversity solely because of marginalized individuals’ unwillingness to embrace their diversity. This would be an incorrect interpretation for a key reason. The reluctance to display marginalized social identity traits occurs in a larger, societal context where such traits are devalued. It is not as though marginalized individuals alone determined that these traits might best be concealed. In fact, as the above discussion reveals and our findings suggest, the display of marginalized social identity traits exposes individuals to a host of negative evaluations, regardless of the race of the evaluator.

### Limitations and Future Research

Several limitations should be considered in light of our study findings. First, all of our studies were experimental in nature. It would be beneficial for future research to determine if our findings replicate in a field setting. For example, it would be fruitful to conduct research with hiring managers and organizational decision makers to determine if hairstyle and evaluator race interact to affect actual recruiting, promotion and retention decisions. Importantly, such behavioral outcomes as recruitment, promotion and retention may be less susceptible to social desirability than the perceptual outcomes measured in the current research. Additionally, our results could be partially explained by white evaluators’ social desirability concerns. It might not be politically correct for white evaluators to express negative reactions to Afrocentric hair. Thus, white individuals may have suppressed their honest negative reactions to the employment candidates with Afrocentric hair. While this is certainly a potential explanation for our findings, we are heartened by the fact that *all* participants, regardless of race, rated Afrocentric hairstyles as less professional than Eurocentric hairstyles. This suggests that the study stimuli were strong enough to at least partially overcome white evaluators’ potential social desirability concerns.

Another study limitation is that the hairstyles used in our studies were simplistic given that both Black hair and White hair may come in a variety of textures and that hair can be styled in a number of ways (e.g., more modern, more traditional, etc.). However, when selecting the hairstyles for our studies we focused on two key elements. First, we selected hairstyles that have been traditionally identified as Black/Afrocentric and White/Eurocentric). We chose this approach because hair is a racial phenotypical trait (Maddox, 2014) and a racial marker used by perceivers to racially categorize targets (MacLin and Malpass, 2001). Thus, it made sense to begin our line of inquiry with hairstyles readily identifiable as Black/Afrocentric and White/Eurocentric. Second, we chose Afrocentric hairstyles (i.e., Afro and Dreadlocks) that reflect how hair naturally grows without much manipulation (other than combing or brushing) or adornment (e.g., beads, decorations, etc.)^6^. We concur that future research should consider a fuller; more nuanced range of hairstyles so that we can better understand the boundary conditions of our study outcomes and the causality in our model. This is particularly relevant given the increasing numbers of Black women who are opting to wear their Afrocentric hair (Mintel, 2014) and the likely proliferation of hairstyle options.

It might be argued that the current research is not generalizable. While we argue that our findings may generalize to identity traits representative of other demographic groups (e.g., yarmulkes, headscarves, facial adornment, nose piercings, gray hair, etc.), future research could explore if reactions to such displays of ethnicity, religion, age, etc., relate to perceptions of dominance and evaluator identity (i.e., in-group versus out-group member). Our hope is that this line of research will provide helpful guidance to organizations as they strive to manage an increasingly diverse workforce.

Finally, future research should more closely examine potential mediating mechanisms between the expression of marginalized identity traits (donning of Afrocentric hair, wearing yarmulkes, etc.) and ratings of professionalism and dominance. For example, what is the role of collective threat in determining in-group member responses to in-group member display of marginalized identity traits? We also recommend the investigation of other potential outcomes of the expression of marginalized identity traits (e.g., self-esteem, performance, interpersonal relationships, professional image, etc.). Further, what are the conditions under which such expression might be empowering and have positive effects versus have negative consequences? We believe that these are fruitful lines of inquiry for future research.

## Conclusion

This research is important to consider given a recent movement amongst Black women to wear their Afrocentric hair instead of chemically processed Eurocentric styles (Mintel, 2014). This trend is evidenced by the continued decline in sales of chemical relaxers (i.e., straighteners) while the number of products available to style Afrocentric hair rise; additionally, the majority of Blacks believe that Afrocentric hairstyles are not merely a fad but an expression that is here to stay (Mintel, 2014). However, if Afrocentric hairstyles are related to dominance perceptions amongst Blacks and lower ratings of professionalism amongst both Black and White individuals, this movement to wear Afrocentric styles may have unintended consequences.

As the number of Black women opting to wear Afrocentric hair continues to rise, organizations may find it increasingly difficult to maintain standards of professionalism that prohibit or discourage Afrocentric hair styles. It is important for organizations to examine what purpose is served by the current culturally determined standards of appearance and how such standards relate to professionalism. Arguably, professionalism should have much less to do with appearance and much more to do with performance. Yet, judgments of executive presence, whether or not an individual is perceived as possessing “what it takes” to succeed (Hewlett, 2014, p. 1), are affected by appearance. In fact, according to a survey of US-based senior leaders, being polished and groomed is the top aspect of appropriate appearance, and unkempt hair is noted as a key female appearance blunder behind poorly maintained clothing (Hewlett, 2014). Important to this discussion, Black women they may tire of dedicating hours of time to coax their naturally kinky textured hair into more acceptable Eurocentric styles (Rosette and Dumas, 2007). Additionally, as Black women witness an increasing number of their counterparts electing Afrocentric hairstyles they may join in and lay down the cognitive and emotional burden of navigating majority-based interpretations of their professionalism.

Our discussion suggests that Black women walk a tightrope as they may desire to wear their Afrocentric hair but fear negative evaluation if they do so. This need not be the case. Ursula Burns, the only Black CEO of a Fortune 500 company, exemplifies how Afrocentric hair does not have to hinder professional success. In fact, we think it plausible that CEO Burn’s authentic expression of her Afrocentric hair coupled with her impeccable pedigree may have helped her achieve status and distinction in the workplace. We believe that CEO Burns’ embrace of her Afrocentric hair conveyed her comfort with her authentic self. Such authenticity has benefits for the individual, as well as, the organization.

Authenticity refers to when internal experiences (e.g., feelings, thoughts) are aligned with external expressions (e.g., verbal disclosures, nonverbal displays, attire, grooming, etc.; Roberts et al., 2009). For individuals, authenticity facilitates positive self-regard (Roberts et al., 2009), adaptive functioning (Kernis and Goldman, 2006); well-being (Wood et al., 2008); and, self-esteem (Kernis, 2003). In contrast, inauthenticity may result in stress, anxiety, identity conflict and low self-esteem (Wood et al., 2008; White and Tracey, 2011). Organizations benefit from having authentic employees by obtaining the positive benefits of authenticity and avoiding the costs of suppression (e.g., emotional exhaustion, intention to leave, Hewlin, 2003, 2009). These arguments suggest that professional standards that prohibit or discourage Afrocentric hair may be ineffective at improving professionalism while hampering employee authenticity and leading to a host of negative outcomes. Organizations must ask if such narrow professional standards are worth the cost. Perhaps if organizations revisit these professional standards and create workplace environments that are more inclusive and truly receptive to marginalized social identity traits, marginalized group members will feel more comfortable expressing their diversity at work and organizations will be the better for it.

## Conflict of Interest Statement

The authors declare that the research was conducted in the absence of any commercial or financial relationships that could be construed as a potential conflict of interest.

## Footnotes

1 For example, Afro hairstyles are considered Afrocentric as they reflect hair textures that naturally occur for the majority of individuals who are of African descent. In contrast, chemically processed, straightened hair is considered Eurocentric as it reflects the straight hair textures of those of European descent.

2 To assess attractiveness, we asked participants, “How attractive is this person”, rated 1 (Very Unattractive), 2 (Unattractive), 3 (Somewhat Unattractive), 4 (Average), 5 (Somewhat Attractive), 6 (Attractive), 7 (Very Attractive).

3 Number of outliers trimmed: three from Study 1 (*N* = 200), six from Study 2 (*N* = 510), one from Study 3 (*N* = 291).

4 We noted that the majority of the failed manipulation checks resulted from two of the White, straight-haired images. These images were not used in subsequent studies.

5 It is important to note that the present studies did not utilize a fully factorial design. We did not have respondents evaluate White women with Afrocentric hair. White women donning Afrocentric hair is a rare occurrence and including this manipulation may have alerted respondents to the purpose of the study.

6 Dreadlocks are a hairstyle that result when hair is initially sectioned into braids/twists and then left undisturbed (i.e., not combed, brushed, etc.) and allowed to grow together.
